# Malocclusion Complexity in Patients with Disc Displacement Disorders: A Case–Control Study

**DOI:** 10.3390/healthcare11152202

**Published:** 2023-08-04

**Authors:** Iván Daniel Zúñiga-Herrera, Fernando Javier Aguilar-Pérez, Mauricio Escoffié-Ramírez, José Rubén Herrera-Atoche

**Affiliations:** Department of Orthodontics, School of Dentistry, Autonomous University of Yucatan, Mérida 97000, Mexico; ivan.zuniga@correo.uady.mx (I.D.Z.-H.); fernando.aguilar@correo.uady.mx (F.J.A.-P.); mauricio.escoffie@correo.uady.mx (M.E.-R.)

**Keywords:** epidemiology, growth and development, orthodontic index, temporomandibular joint disorders

## Abstract

This study aimed to determine the possible association between disc displacement (DD) disorders and malocclusion complexity. This cross-sectional study was carried out using a case–control design. The Research Diagnosis Criteria for Temporomandibular Disorders were used to identify cases and controls. The Index of Complexity, Outcome, and Need (ICON) was used to quantify malocclusion complexity as easy, mild, moderate, difficult, or very difficult. A total of 310 subjects were included: 130 cases and 180 controls. A binary logistic regression (*p* < 0.05) was used to identify associations. The odds ratio (OR) was also calculated. DD was associated with sex, age, and malocclusion complexity (*p* < 0.05). The malocclusion complexity comparison showed that 89.3% of the controls fell within the easy–moderate levels of the ICON, whereas 85.4% of the cases were in the moderate–very difficult levels (*p* ≤ 0.001). Difficult and very difficult malocclusions had high ORs (9.801 and 9.689, respectively) compared to the easy cases. In conclusion, patients with malocclusion complexity levels classified as difficult or very difficult have greater odds of presenting DD.

## 1. Introduction

Disc displacement (DD) is the most common internal derangement affecting the temporomandibular joint. DD prevalence has been reported between 18% to 35% in the general population. This condition usually starts during childhood and adolescence [1] and reaches its maximum prevalence in the fourth decade of life [2]. A clicking sound on the joint is the classical sign of this condition, sometimes accompanied by pain [1].

Another symptom of DD is the limitation of some mandibular movements since this condition can block the translation of the condyles, creating a close lock. Most of the time, the close lock is symptom-free, and only in rare cases is it accompanied by pain and limited mouth opening. Given that only cases with signs and symptoms search for treatment, there is a misconception that the close lock of the temporomandibular joint usually is painful and produces some degree of disability. However, in many cases, it tends to correct itself within months; therefore, the treatment is usually conservative [1].

Temporomandibular disorder (TMD) etiology is multifactorial. Environmental factors such as sociodemographics, general health status, pain sensitivity, psychological variables, and orofacial characteristics influence the onset of TMD differently [3,4]. Among those environmental factors, some stand out; for example, in the sociodemographic group, age and sex are the highlights. TMD signs and symptoms have two major age clusters, the first between 30 and 35 years of age, where myofascial pain and DD are frequently found together. The second cluster is between 50 and 55 years of age; in this case, myofascial pain is usually accompanied by arthritic disorders [2]. As for sex, females are more frequently affected by TMD, which is valid for any disorders in the TMD spectrum [5].

In the next category, it has been proved that TMD progresses disproportionately in individuals with poor general health. Smoking, poor sleep quality, and some comorbid diseases are associated with TMD [3].

Pain sensitivity and psychological factors are present in many TMD patients. While somatic symptoms are strong predictors for TMD, it is also true that patients affected by chronic TMD usually have low pain thresholds as a consequence; therefore, care should be taken with this complex symptomatology. When psychological variables are studied in relation to TMD, depression is strongly associated with its symptoms, although other variables like anxiety and somatization have been widely studied [3,4].

The last environmental group is orofacial characteristics, which include anatomical factors, dental occlusion, parafunctions like bruxism, and a history of oral trauma. While it is clear that trauma [1,6] and parafunctions [3] are strongly related to the onset of TMD, anatomical factors and dental occlusion are more controversial [1]. Scientific evidence shows that dental malocclusion is not an etiological factor for the presence of TMD [7]. However, there is evidence that patients with complex malocclusions have higher odds of presenting signs and symptoms of TMD [8]. Lastly, some anatomical variations are frequently present in subjects affected by TMD. [1]. 

On the other hand, there are genetic issues [3,9]. For example, COL5A1 rs12722 polymorphism has been identified as a risk factor for disc displacement without reduction in the Polish Caucasian population [9]. Conversely, combined polymorphisms increase the risk of presenting some forms of TMD. Large-scale studies such as the OPPERA project (Orofacial Pain: Prospective Evaluation and Risk Assessment) found that biopsychosocial risk factors define distinct clusters of people with (or at risk of) TMD [3].

Considering that TMD includes different conditions, it is essential to analyze how each of them is affected by biopsychosocial factors. A standardized way to do that is using the Research Diagnostic Criteria for Temporomandibular Disorders (RDC/TMD), which consider the most relevant etiological factors [10,11]. The RDC/TMD are divided into two axes. Axis I gives the diagnosis of the subject evaluated, classified into three different groups: Group I myofascial disorders (myofascial pain and myofascial pain with limited opening), Group II disc displacement disorders (disc displacement with reduction, disc displacement without reduction with limited opening, and disc displacement without reduction without limited opening), and Group III arthritic disorders (arthralgia, osteoarthritis, and osteoarthrosis). Any given patient could have single, double, or triple diagnoses. On the other hand, Axis II gives information regarding psychological status and pain-related disability. Through this axis, it is possible to assess essential factors such as the grade of pain, the presence of depression, and bruxism. Therefore, the RDC/TMD give a complete evaluation since they are consistent with the biopsychosocial health model [11].

Like any other TMD, disc displacement disorder etiology is multifactorial, with many associated variables. Age [2] and sex [5] (females) are strongly related to disc displacement. Unlike myofascial pain and arthritic conditions, disc displacement disorders do not strongly relate to psychological factors [12]. However, the role of other variables such as anatomy, oral parafunctions, generalized joint hypermobility, changes in joint lubrication, history of trauma [1], and some types of malocclusions in disc displacement disorders remains controversial.

For many years, the scientific community has debated the role of occlusion as an etiological factor for TMD [7] and disc displacement disorders [13]. It was thought that occlusal interferences during mandibular movements could be a trigger. Therefore, the absence of canine or incisal guidance was considered an etiological factor. However, evidence has shown no cause–effect relationship between disc displacement and occlusal features [13,14]. Furthermore, there is proof that occlusal features have a low predictive value for detecting disc displacement [13]. Nonetheless, some studies have found evidence associating disc displacement and specific malocclusions, such as hyper-divergent class II [15,16,17,18], class III asymmetrical malocclusions [19], and unilateral crossbites [20]. Although no causal relationship has been established, these associations are well documented and prove a relationship between complex malocclusions and disc displacement.

Since malocclusion combines many traits, it has recently been suggested that it should be studied with a comprehensive vision of the complex phenomenon it is [8]. In this regard, evidence shows that high levels of malocclusion complexity are accompanied by higher odds of presenting with TMD signs or symptoms [8]. The novelty of this study is its approach, which considers malocclusion as a complex phenomenon instead of isolated traits and studies its association with the presence of DD.

This study aimed to determine the possible association between disc displacement and malocclusion complexity.

## 2. Materials and Methods

This cross-sectional study was conducted using a case–control design. The inclusion criteria considered subjects of either sex with permanent dentition who had not undergone orthodontic treatment. The exclusion criteria included subjects under anti-inflammatories or analgesics coinciding with the time of the evaluation and patients with extensive dental restorations. The study subjects were patients from a dental school. All subjects participating in this study gave written informed consent, and a research ethics committee approved the study (CIRB-2017-004).

This study’s sample estimation considered a case–control match with an odds ratio (OR) of 2, 80% of power (α = 0.05), and 61% of the cases exposed to some malocclusion trait [21]. In the end, 375 patients were assessed. The diagnosis of TMD was made using Axis I of the RDC/TMD following the published protocol [10,11]. The patients diagnosed with disc displacement (with or without reduction) in one or both joints were classified as cases. Since muscular or articular disorders usually accompany disc displacement, the patients with mouth-opening limitations of muscular origin or arthralgia were eliminated to avoid bias. However, given that myofascial pain is the most frequent disorder found with disc displacement, it was decided to make two models. The first model included patients with single or combined diagnoses (disc displacement with or without myofascial pain without mouth-opening limitations) in the case group. The second model only allowed disc displacement disorder cases (single diagnosis). The patients who had received previous orthodontic treatment or had restorations that might affect their Index of Complexity, Outcome, and Need (ICON) score evaluation were also excluded. The subjects without signs or symptoms of TMD were used as controls. This process gave a total of 130 cases and 180 controls.

An even age distribution across the cases and controls was accomplished by dividing them into percentiles. The age groups were as follows: group A, ≤17 years; group B, 18 to 21 years; group C, 22 to 28 years; and group D, ≥29 years.

The depression level (normal, moderate, or severe) and the presence of bruxism were estimated using Axis II of the RDC/TMD. For the cases, the grades of chronic pain were calculated based on Axis II of the RDC/TMD:

- Low disability with low intensity (grade I)
- Low disability with high intensity (grade II)
- High disability with moderate limitation (grade III)
- High disability with severe limitation (grade IV)

The level of malocclusion complexity was calculated for both groups using the ICON [22,23]. This index includes five components, and each one gives a raw score multiplied by a specific weight that, when added together, yields a final score for a particular patient. The components are described below:

- The first is the dental aesthetics component, which uses the same dental aesthetic component of the Index of Orthodontic Treatment Need (IOTN) developed by Shaw et al. (1991) [24], and it uses an illustrated scale for its evaluation graded from 1 to 10 from the most to the least attractive dental arrangement.
- The second component is dental crossbite. A patient is considered to have dental crossbite in the posterior segment if one or more teeth are in a cusp-to-cusp relationship. In the anterior segment, the guideline is the presence of an edge-to-edge relationship. Subjects with anterior and posterior crossbites will have higher values for this component.
- The third component is the anterior vertical relationship. This trait considers the open-bite and deep-bite cases. For both traits, the index only scores the tooth with the highest value; in cases that present both, the worst one is to be taken.
- The fourth component is upper-arch crowding/spacing. This component evaluates the discrepancy between the mesiodistal crown diameters against the available arch circumference. This trait does not include estimation for the curve of Spee. The presence of an impacted tooth immediately gives the maximum score for this category.
- The fifth component is the buccal-segment antero-posterior relationship. The molar, premolar, and canine sagittal relationships are considered for this variable. To calculate this trait, both sides of the occlusion are evaluated and added together.

Given the combination of all these criteria, the index expresses the degree of malocclusion complexity because it includes the esthetic, transversal, sagittal, and vertical dimensions, which is why it was the index of choice for this study. According to their ICON score, the patients’ malocclusions were classified into the following categories:

- Easy (<29);
- Mild (29–50);
- Moderate (51–63);
- Difficult (64–77);
- Very difficult (>77).

### 2.1. Method of Error

A single operator, previously calibrated (kappa RDC/TMD: 0.84, ICON: 0.94), carried out the RCD/TMD questionnaires and physical examinations.

### 2.2. Statistical Analysis

The statistical analysis was conducted using SPSS software (version 20). For both models, a bivariate analysis (chi-square test) was performed to identify associations between DD and sex, age, malocclusion complexity (ICON), depression, and bruxism (*p* < 0.05). Subsequently, a binary logistic regression was performed, including the variables with *p* < 0.2 in the bivariate analysis (Nagelkerke’s R2 coefficient and Hosmer–Lemeshow goodness of fit were calculated). Ultimately, confidence intervals (CIs) of 95% and ORs were estimated for both tests (chi-square and binary logistic regression).

To determine the model that best describes the relationship between the variables, the goodness of fit and the Nagelkerke test results were compared. Biological factors were also considered as selection criteria for the best model.

## 3. Results

The final sample was composed of 310 subjects (130 cases and 180 controls). The mean age of the sample was 24.94 +/− 11.19 years old, 35.2% were males (*n* = 109), and 64.8% were females (*n* = 201). Concerning malocclusion complexity, 18.4% (*n* = 57) of the individuals fell in the easy level, 16.1% (*n* = 50) were mild, 33.2% (*n* = 103) were moderate, 22.6% (*n* = 70) were difficult, and 9.7% (*n* = 30) were very difficult. As for depression, 36.8% (*n* = 114) of the subjects were at the moderate level, and 10% (*n* = 31) were severe; the remaining 53.2% (*n* = 165) were considered normal. According to the RCD/TMD, 47.7% (*n* = 148) of the subjects were bruxist.

Regarding the case group, 91.54% (*n* = 119) of the patients had disc displacement with reduction, 2.31% (*n* = 3) had disc displacement without reduction and without mandibular limitation, and 6.15% (*n* = 8) had disc displacement without reduction but with mandibular limitation. Given the small number of subjects affected by disc displacement without reduction (*n* = 11), it was decided to combine all cases and compare them against the controls as a single group.

Thirty case-group individuals (23.07%) had dual diagnoses (disc displacement and myofascial pain without mouth-opening limitation).

### 3.1. First Model (Cases with Single or Combined Diagnoses)

#### 3.1.1. Bivariate Analysis

**Figure 1:**
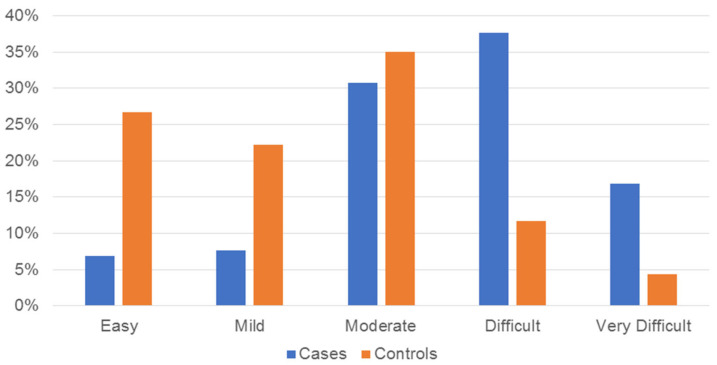
Bar chart showing the distribution of cases and controls of the first model (includes cases with single or combined diagnoses) according to the Index of Complexity Outcome and Need levels and expressed in percentages.

Concerning sex, although 70.8% of the cases were females (*n* = 92), no statistically significant association was found between disc displacement and being female (*p* = 0.063). Regarding age, 53.1% (*n* = 69) of the cases were in group D, whereas 50.6% (*n* = 91) of the controls were in groups B (*n* = 46) and C (*n* = 45) (*p* < 0.001). The malocclusion complexity comparison showed that 89.3% (*n* = 151) of the controls were spread within the lower three levels of the ICON (easy–moderate), whereas 85.4% (*n* = 111) of the cases were in the higher three levels (moderate–very difficult); this finding was statistically significant (*p* < 0.001) (Figure 1). Most cases showed moderate or severe depression, while most controls were classified as normal; this comparison had a statistically significant outcome (*p* = 0.002). Bruxism showed no significant association with disc displacement (*p* = 0.806). The results of the bivariate test for the first model are shown in Table 1.

As for the grade of chronic pain in this model, 40.8% (*n* = 53) of the cases were classified as grade I, 10.8% (*n* = 14) as grade II, and 3.1% (*n* = 4) as grade III; 45.4% (*n* = 59) of the cases were pain-free, and none was classified as grade IV.

#### 3.1.2. Binary Logistic Regression

This analysis showed a significant association between disc displacement and the following factors: sex, age, and malocclusion complexity. According to the test results, females had a 1.8 OR (*p* = 0.046). Patients in age group D had an OR of 2.706 (*p* = 0.004). Regarding malocclusion complexity, the OR for the moderate group was 2.701 (*p* = 0.026), and it increments to 9.801 (*p* < 0.001) and 9.689 (*p* < 0.001) for the difficult and very difficult groups, respectively. No significant association was found between disc displacement and depression. The Hosmer–Lemeshow goodness-of-fit test result was *p* = 0.843. Finally, the Nagelkerke (R2) value was 0.315. All data regarding the binary logistic regression for the first model can be seen in Table 2.

### 3.2. Second Model (Cases with Single Diagnosis)

#### 3.2.1. Bivariate Analysis

**Figure 2:**
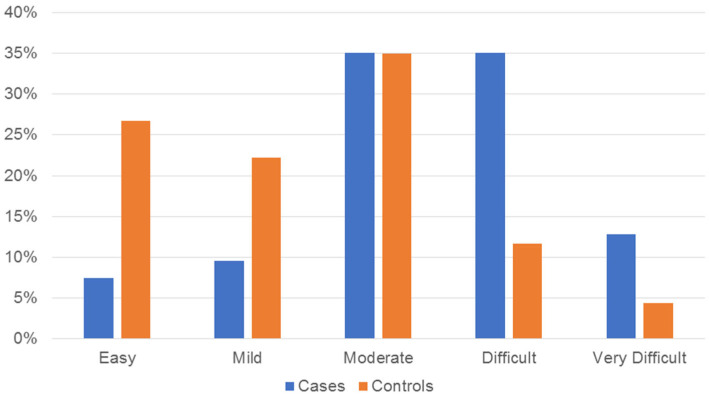
Bar chart showing the distribution of cases and controls of the second model (includes cases with single diagnosis) according to the Index of Complexity Outcome and Need levels and expressed in percentages.

No statistically significant association was found between disc displacement and sex (*p* = 0.293). Regarding age, 35.1% (*n* = 33) of the cases were in group D, whereas 36.2% (*n* = 34) of the controls were in groups B (*n* = 17) and C (*n* = 17) (*p* < 0.03). The malocclusion complexity comparison showed that 83.9% (*n* = 151) of the controls were spread within the lower three levels of the ICON (easy–moderate), whereas 83% (*n* = 78) of the cases were in the higher three levels (moderate–very difficult); this finding was statistically significant (*p* < 0.001) (Figure 2). Bruxism and depression showed no significant association with disc displacement (*p* = 0.458 and *p* = 0.182, respectively). The results of the bivariate test for the second model are shown in Table 3.

As for the grade of chronic pain in this model, 34.04% (*n* = 32) of the cases were classified as grade I, 2.13% (*n* = 2) as grade II, and 1.06% (*n* = 1) as grade III; 62.77% (*n* = 59) of the cases were pain-free, and none was classified as grade IV.

#### 3.2.2. Binary Logistic Regression

This analysis showed a significant association between disc displacement and malocclusion complexity. According to the test results, a moderate malocclusion complexity level showed a 3.371 OR value (*p* = 0.012), and it increments to 10.411 (*p* < 0.001) and 9.357 (*p* = 0.001) for the difficult and very difficult groups, respectively. No significant association was found between disc displacement with age or depression. The Hosmer–Lemeshow goodness-of-fit test result was *p* = 0.49. Finally, the Nagelkerke (R2) value was 0.202. All data regarding the binary logistic regression can be seen in Table 4.

### 3.3. Model Selection

Both models showed a significant association between malocclusion complexity and the presence of disc displacement disorders. The first model was kept because of better goodness of fit (first model: 0.843; second model: 0.49) and the Nagelkerke test (first model: 0.315; second model: 0.202) results, as well as because of a realistic biological perspective, since sex and age are well-known risk factors for disc displacement disorders, and myofascial pain is a usual concomitant condition.

## 4. Discussion

Binary logistic regression analysis showed a significant association between disc displacement and the following factors: sex, age, and malocclusion complexity. The literature demonstrates females are more likely to present with TMD [5]. In the particular case of disc displacement disorders, some authors explain this phenomenon as an altered collagen metabolism combined with joint laxity of genetic origin [25].

Concerning age, the results of this study show that patients over 29 years of age have a higher risk of disc displacement. This finding concurs with evidence that TMD signs and symptoms have their first peak around 30–35 years of age, and this peak is mainly composed of disc displacement disorders accompanied by muscle disorders [2].

Depression was associated with disc displacement in the bivariate analysis. However, the logistic regression test showed no relationship between depression and disc displacement. It is essential to understand that many pathologies coexist in TMD patients. In this study, 23.07% of the case group patients had dual diagnoses, and myofascial pain strongly correlates with depression [26,27,28,29], anxiety [28,30], and somatization [27]. Although depression is more strongly associated with myofascial pain with mouth-opening limitations [26], and those subjects were eliminated from this study, the presence of myofascial pain in some cases may influence the bivariate analysis results, so care must be taken when drawing conclusions. Regarding pain, 54.6% of the patients in the case group experienced some pain. However, 40.8% of the cases experienced only grade-I-level pain, consistent with the evidence that pain is not a significant issue with this condition, which usually resolves itself within months [1].

Regarding malocclusion complexity, many studies have reported that patients affected by disc displacement with or without reduction tend to have class II malocclusion [16,18,31], a hyperdivergent growth pattern [15,16,17,18], and a retrognathic mandible [15,17,18].

In 2021, John et al. [32] compared Angle’s class II vertical patients with class II horizontal cases and class I subjects. They found that class II vertical patients are more susceptible to developing TMD [32]. The combination of two traits (class II and hyperdivergent growth pattern) has the highest risk of showing maximum alterations in the disk position [33]. On the other hand, it has been shown that the tendency toward a high mandibular angle increases with the severity of the disc displacement disorder (disc displacement without reduction) [15,17,18,34] regardless of whether the patient is symptomatic [17].

Other authors have reported that disc displacement without reduction is associated with severe cases such as skeletal class II patients with open bites or skeletal class III subjects with asymmetries [19]. Scientific evidence demonstrates the association between disc displacement and those morphologic traits [15,16]. Even though dental occlusion is not an etiological factor for TMD [7,35], all the previously mentioned characteristics (skeletal class II, asymmetric prognathism, hyperdivergent growth pattern, and open bite) increase the orthodontic treatment difficulty. Therefore, the odds of seeing high levels of malocclusion complexity are increased in patients with these characteristics.

Many papers have explored the predictive value of occlusal features or dental malocclusion traits for disc displacement disorders, and their results have shown a low predictive value for this condition [13,14,36]. This study found high ORs for difficult (9.801) and very difficult (9.689) malocclusions compared with easy cases, which concurs with the evidence of the association of complicated cases (class II hyperdivergent, open bite, and class III asymmetric) with the presence of disc displacement disorders. Hence, given that malocclusion is a complex condition with many traits co-existing simultaneously, these results suggest a predictive value from a comprehensive approach to assessing malocclusions.

### Limitations

To avoid bias, patients with arthralgia or myofascial pain with mouth-opening limitations were excluded from the study. However, the results of this study should be seen in light of its limitations. For example, it is important to note that the patients in the case group were included regardless of whether they had one or both joints affected by DD. Also, bruxism and depression were evaluated using TMD/RDC Axis II, and the results may vary with other instruments.

Another consideration is that temporomandibular disorders (muscular, disc displacement, and arthritis) usually occur combined (dual or triple diagnoses). In this study, the main confounding factor was the presence of disc displacement with myofascial pain in some patients. The authors developed two models to address the issue, the first including single-diagnosis patients and the second with dual-diagnosis cases. As explained before, the first model was chosen based on the goodness of fit (first model: 0.843; second model: 0.49) and the Nagelkerke test (first model: 0.315; second model: 0.202) results; biological criteria were also considered. However, be aware that other confounders not considered in this study may be present in any clinical situation, for example, genetical factors, and other variables considered in the biopsychosocial health model such as sociodemographic factors, general health status, pain sensitivity, and other psychological conditions (anxiety or somatization). Finally, it must be stated that this is a cross-sectional study with a limited number of participants, so no generalizations should be made.

## 5. Conclusions

The results of this study demonstrate that sex, age, and malocclusion complexity are associated with disc displacement disorders. Patients with malocclusion complexity levels classified as difficult or very difficult have greater odds of presenting disc displacement disorders.

## Figures and Tables

**Table 1:** First model bivariate analysis (cases with single or combined diagnoses included).

Variables		Cases		Controls		*p*	OR	95% CI	
		%	(*n* = 130)	%	(*n* = 180)			Lower	Upper
**Sex**	Male	29.2	38	39.4	71	0.063	1.577	0.974	2.553
	Female	70.8	92	60.6	109				
**Age**	Up to 17	20.8	27	30	54	<0.001 *			
	From 18 to 21	13.1	17	25.6	46				
	From 22 to 28	13.1	17	25	45				
	29 or more	53.1	69	19.4	35				
**ICON**	Easy	6.9	9	26.7	48	<0.001 *			
	Mild	7.7	10	22.2	40				
	Moderate	30.8	40	35	63				
	Difficult	37.7	49	11.7	21				
	Very Difficult	16.9	22	4.4	8				
**Bruxism**	No	53.1	69	51.7	93	0.806	0.945	0.602	1.485
	Yes	46.9	61	48.3	87				
**Depression**	No	42.3	55	61.1	110	0.002 *			
	Moderate	43.2	56	32.2	58				
	Severe	14.6	19	6.7	12				

(*) Statistically significant; OR: odds ratio; CI: confidence interval.

**Table 2:** First model binary logistic regression (cases with single or combined diagnoses included).

Variables		*p*	OR	95% CI	
				Lower	Upper
**Sex**	Male †				
	Female	0.046 *	1.8	1.011	3.204
**Age**	Up to 17 †	0.006 *			
	From 18 to 21	0.872	0.937	0.421	2.083
	From 22 to 28	0.969	0.984	0.435	2.225
	29 or more	0.004 *	2.706	1.378	5.312
**ICON**	Easy †	<0.001 *			
	Mild	0.677	1.246	0.442	3.515
	Moderate	0.026 *	2.701	1.123	6.493
	Difficult	<0.001 *	9.801	3.737	25.705
	Very Difficult	<0.001 *	9.689	2.756	34.06
**Depression**	No †	0.707			
	Moderate	0.553	0.831	0.451	1.532
	Severe	0.736	1.179	0.453	3.065

(†) Reference category; (*) statistically significant; OR: odds ratio; CI: confidence interval. Hosmer–Lemeshow goodness-of-fit test: *p* = 0.843. Nagelkerke R2: 0.315.

**Table 3:** Second model bivariate analysis (includes cases with single diagnosis).

Variables		Cases		Controls		*p*	OR	95% CI	
		%	(*n* = 94)	%	(*n* = 180)			Lower	Upper
**Sex**	Male	33	31	39.4	71	0.293	1.324	0.784	2.235
	Female	67	63	60.6	109				
**Age**	Up to 17	28.7	27	30	54	0.03 *			
	From 18 to 21	18.1	17	25.6	46				
	From 22 to 28	18.1	17	25	45				
	29 or more	35.1	33	19.4	35				
**ICON**	Easy	7.4	7	26.7	48	<0.001 *			
	Mild	9.6	9	22.2	40				
	Moderate	35.1	33	35	63				
	Difficult	35.1	33	11.7	21				
	Very Difficult	12.8	12	4.4	8				
**Bruxism**	No	56.4	53	51.7	93	0.458	0.827	0.501	1.366
	Yes	43.6	41	48.3	87				
**Depression**	No	51.1	48	61.1	110	0.182			
	Moderate	37.2	35	32.2	58				
	Severe	11.7	11	6.7	12				

(*) Statistically significant; OR: odds ratio; CI: confidence interval.

**Table 4:** Second model binary logistic regression (includes cases with single diagnosis).

Variables		*p*	OR	95% CI	
				Lower	Upper
**Age**	Up to 17 †	0.76			
	From 18 to 21	0.934	0.967	0.437	2.14
	From 22 to 28	0.947	1.028	0.456	2.319
	29 or more	0.354	1.402	0.686	2.865
**ICON**	Easy †	<0.001 *			
	Mild	0.451	1.528	0.508	4.596
	Moderate	0.012 *	3.371	1.307	8.697
	Difficult	<0.001 *	10.411	3.739	28.991
	Very Difficult	0.001 *	9.357	2.435	35.952
**Depression**	No †	0.626			
	Moderate	0.5	0.806	0.432	1.507
	Severe	0.643	1.261	0.473	3.366

(†) Reference category; (*) statistically significant; OR: odds ratio; CI: confidence interval. Hosmer–Lemeshow goodness-of-fit test: *p* = 0.49. Nagelkerke R2: 0.202.

## Data Availability

The data that support the findings of this study are available from the corresponding author upon reasonable request.
